# Painful tongue leiomyoma

**DOI:** 10.1016/S1808-8694(15)31032-6

**Published:** 2015-10-19

**Authors:** Felippe Felix, Geraldo Augusto Gomes, Shiro Tomita, Albino Fonseca Júnior, Luzia Abrão El Hadj Miranda, Andréia Migueres Arruda

**Affiliations:** a MS Student - Federal University of Rio de Janeiro.; b MS Student - Federal University of Rio de Janeiro.; c Full Professor, Head of the Otorhinolaryngology Department - Clementino Fraga Filho University Hospital - Federal University of Rio de Janeiro.; d Pathology 3rd year Resident - Clementino Fraga Filho University Hospital - Federal University of Rio de Janeiro.; e MD. Otorhinolaryngologist - Clementino Fraga Filho University Hospital - Federal University of Rio de Janeiro; f MD. 3rd year Otorhinolaryngology Resident - Federal University of Rio de Janeiro. Universidade Federal do Rio de Janeiro.

**Keywords:** benign mass, leiomyoma, tongue

## INTRODUCTION

Leiomyomas are benign tumors that originate on the smooth muscle, usually found in the uterus, gastrointestinal tract and skin. This type of tumor rarely occurs in the oral cavity, specially because of the lack of smooth muscle in this area^1^. Its most frequent location in this region, by decreasing order of appearance is: lips, palate, tongue and oral mucosa^2^. It is usually asymptomatic; however it may be painful in some cases.

Our goal with this paper is to report on a case of a patient with a painful tongue leiomyoma.

## CASE REPORT

A 34 year old man, with the Acquired Immunedeficiency Syndrome (AIDS), visited the physician, complaining of a spontaneous stinging pain in the oral cavity for 2 years. During exam we saw a reddish mass, of regular contours, measuring about one centimeter in diameter, located on the tongue base. Tecnecium-99 scintigraphy used to assess the presence of ectopic thyroid tissue was negative.

**Figure 1:**
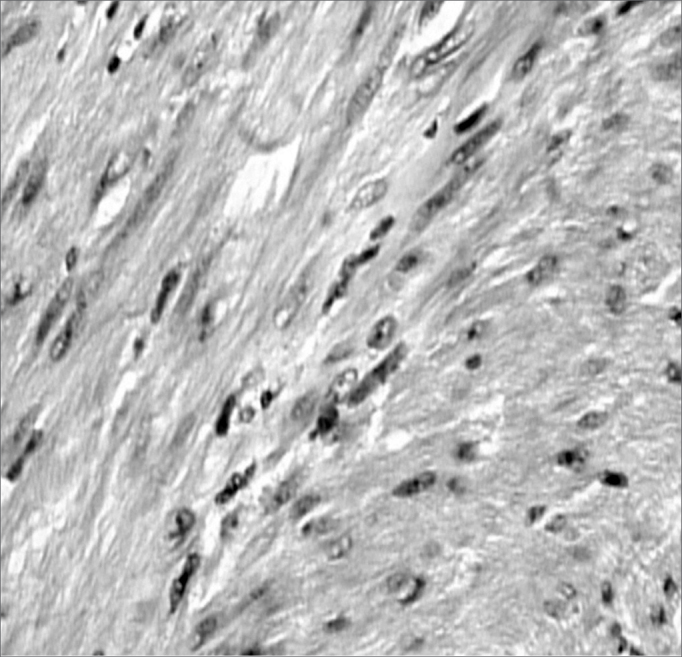
Histopathology confirming it to be a leiomyoma with prolipheration of elongated cells with fasciculated and other times stirred arrangement (100X magnification- Dyed by Hematoxylin & eosin).

The tumor was biopsied and the pathology (Figure 1) showed a solid type of leiomyoma with positive immunohistochemistry for desmine and actine. The lesion was removed through the mouth. The pain improved in the post-operative and the one year follow up did not show recurrence.

## DISCUSSION

Leiomyomas may be divided according to the World Health Organization in three types: angioleiomyomas, solid leiomyomas and leiomyoblastomas. The most common type in the oral cavity is the angioleiomyoma, with 74% prevalence rate, followed by the solid leiomyoma, the most common in the tongue, with 25%3 prevalence. The smooth muscle tumor origin in the oral cavity could be in the middle layer of the arteries, ectopic smooth muscle tissues or the tongue taste buds^2^.

Its occurrence on the tongue was reported in only 23 cases in the English literature^2^. Its color varies from reddish to bluish, depending on its vascularization. Its painful tongue location is rare, with only one case reported in the literature^4^. The pain mechanism is not clear; it is believed to be caused by muscle contractions over the tumor vascularization, causing ischemia^4^. As clinical and histopathology differential diagnosis we have: leiomyosarcoma, fibroma, neurofibroma and lipoma.

We did not find other cases of painful leiomyomas in AIDS patients.

## FINAL COMMENTS

Tongue base leiomyoma differential diagnosis must be carried out bearing in mind the other tumors that may affect this region, and be specially based on histopathology. Pain, although rare, may be its major manifestation, such as the one reported here. This is its first publication of an occurrence in an AIDS patient.
